# Stromal fibroblasts present in breast carcinomas promote tumor growth and angiogenesis through adrenomedullin secretion

**DOI:** 10.18632/oncotarget.14999

**Published:** 2017-02-02

**Authors:** Zohra Benyahia, Nadège Dussault, Mylène Cayol, Romain Sigaud, Caroline Berenguer-Daizé, Christine Delfino, Asma Tounsi, Stéphane Garcia, Pierre-Marie Martin, Kamel Mabrouk, L’Houcine Ouafik

**Affiliations:** ^1^ Aix Marseille Univ, The Institut National pour la Recherche Médicale, Centre de Recherche en Oncologie et Oncopharmacologie, UMR 911, 13005, Marseille, France; ^2^ Assistance Publique Hôpitaux de Marseille, Laboratoire d’Anatomie Pathologique, 13015, Marseille, France; ^3^ Aix Marseille Univ, CNRS, ICR, UMR 7273 CROPS, 13397, Marseille, France; ^4^ Assistance Publique Hôpitaux de Marseille, Service de Transfert d’Oncologie Biologique, 13015, Marseille, France

**Keywords:** adrenomedullin, breast cancer, myofibroblasts, invasion, tumor growth

## Abstract

Tumor- or cancer-associated fibroblasts (TAFs or CAFs) are active players in tumorigenesis and exhibit distinct angiogenic and tumorigenic properties. Adrenomedullin (AM), a multifunctional peptide plays an important role in angiogenesis and tumor growth through its receptors calcitonin receptor-like receptor/receptor activity modifying protein-2 and -3 (CLR/RAMP2 and CLR/RAMP3). We show that AM and AM receptors mRNAs are highly expressed in CAFs prepared from invasive breast carcinoma when compared to normal fibroblasts. Immunostaining demonstrates the presence of immunoreactive AM and AM receptors in the CAFs (n = 9). The proliferation of CAFs is decreased by anti-AM antibody (αAM) and anti-AM receptors antibody (αAMR) treatment, suggesting that AM may function as a potent autocrine/paracrine growth factor. Systemic administration of αAMR reduced neovascularization of *in vivo* Matrigel plugs containing CAFs as demonstrated by reduced numbers of the vessel structures, suggesting that AM is one of the CAFs-derived factors responsible for endothelial cell-like and pericytes recruitment to built a neovascularization. We show that MCF-7 admixed with CAFs generated tumors of greater volume significantly different from the MCF-7 xenografts in nude mice due in part to the induced angiogenesis. αAMR and AM_22-52_ therapies significantly suppressed the growth of CAFs/MCF-7 tumors. Histological examination of tumors treated with AM_22-52_ and aAMR showed evidence of disruption of tumor vasculature with depletion of vascular endothelial cells, induced apoptosis and decrease of tumor cell proliferation. Our findings highlight the importance of CAFs-derived AM pathway in growth of breast carcinoma and in neovascularization by supplying and amplifying signals that are essential for pathologic angiogenesis.

## INTRODUCTION

Although tumorigenesis has classically been viewed as a largely cell-autonomous process involving genetically transformed cancer cells, the importance of stromal cell types populating the neoplastic microenvironment is now well accepted [1, 2]. The contribution of the stromal microenvironment to the development of a wide variety of tumors has been supported by the use of experimental mouse models of cancer pathogenesis [3] and by clinical evidence [4, 5]. The accumulated evidence indicates that tumor cells actively recruit stromal cells, such as inflammatory cells, vascular cells, and fibroblasts [6, 7], into the tumor, and that this recruitment is essential for the generation of a microenvironment that actively fosters tumor growth.

The reactive tumor stroma is characterized by expansion and activation of the fibroblast population, excessive production of extracellular matrix (ECM), and persistant inflammation [7]. The cancer-associated fibroblasts (CAFs) are phenotypically and functionally distinguishable from their normal counterparts in their increased rate of proliferation and differential expression of extracellular matrix (ECM) components and growth factors [7, 8]. Several studies have demonstrated that normal fibroblasts have a role in maintaining epithelial homeostasis by suppressing proliferation and oncogenic potential of adjacent epithelia [3, 9]. However, following neoplastic transformation of epithelia, CAFs have been shown to promote tumor growth by inducing angiogenesis, recruiting bone marrow-derived endothelial progenitor cells, and remodeling the ECM [6, 10, 11]. Interestingly, CAFs can even mediate resistance to antiangiogenic therapy [12]. Some CAFs are related to myofibroblasts, an activated form of fibroblast that plays an important role in wound healing and is characterized by expression of α-SMA. Not all CAFs, however, express α-SMA. Increasingly, fibroblasts in tumor tissues are being recognized as a diverse population of myofibroblastic cells intermixed with other fibroblastic cells that do not express α-SMA but may nevertheless be tumor promoting [13, 14].

Characterization of the expression profiles of CAFs has identified this cell type as an important producer of chemokines and growth factors [10, 11]. Adrenomedullin (AM), one such factor, belongs to a family of peptides that includes calcitonin, α- and β-calcitonin gene related peptide (CGRP) and amylin. It acts through the G protein-coupled receptor calcitonin receptor-like receptor (CLR), with specificity for AM being conferred by the receptor activity modifying protein 2 (RAMP2) and 3 (RAMP3) [15]. The ability of CLR/RAMP2 and CLR/RAMP3 to respond with high affinity to AM implies the existence of two molecularly distinct AM receptors referred to as AMR_1_ and AMR_2_ receptors [16]. Many functions have been ascribed to AM. It has been shown to be a multifunctional peptide with properties ranging from inducing vasorelaxation to acting as a regulator of cellular growth [17, 18]. AM is widely expressed in a variety of tumor types [19] and was shown to be mitogenic for many human cancer cell lines *in vitro* [20]. Several *in vivo* studies have shown a regression of tumor growth upon the treatment with neutralizing AM antibodies [21–23], AM receptor antagonist [24, 25], or AM receptor interference [26].

It is important to point out that AM from sources other than the tumor cells themselves (i.e., paracrine sources, such as fibroblasts, blood vessels, immune cells, that surround the tumor bed) could influence the behavior of tumor cells. We are gradually beginning to understand the importance of non-tumor cells in the development of cancer [2], but more attention is needed in understanding how it relates to AM production. Accumulating studies suggest a new role for AM as a cross-talk molecule that integrates tumor and tumor-infiltrating mast cells [27], tumor-infiltrating macrophages [28], or endothelial cells of the tumor [29] communication, underlying a promotion mechanism to facilitate angiogenesis and tumor growth. These results provide a new insight into the dynamic nature of these tumor-infiltrating cells during the tumor growth and support that AM can function as a key factor in this process. Many reports suggest that fibroblasts in tumor masses possess biological characteristics distinct from those of normal fibroblasts [10, 11]. In this study, characterization of human breast carcinomas CAFs led to the identification of AM as a novel CAF-derived tumor stimulatory factor that played a determinant role in human breast cancer, especially with respect to growth, invasion and angiogenesis.

## RESULTS

### Isolation of primary fibroblastic population from invasive human breast cancers

**Figure 1:**
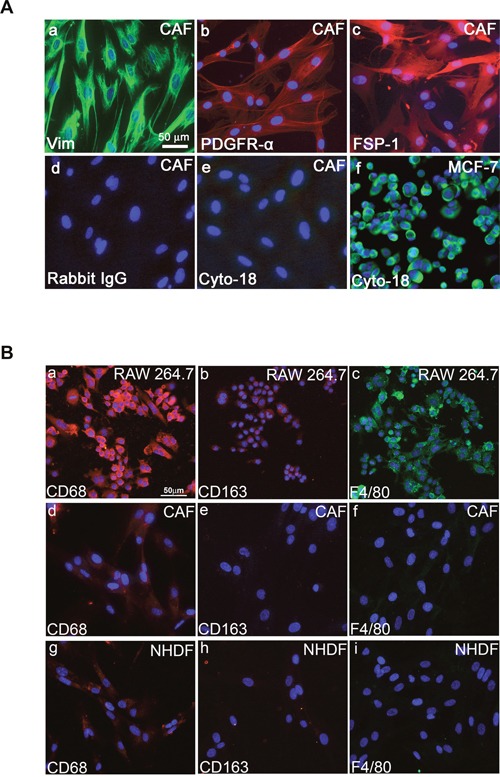
Fibroblastic properties of primary human fibroblasts prepared from human breast cancer tissues **A**. Immunofluorescent staining of cultured CAFs **(a, b, c, d**, and **e)** and MCF-7 cells **(f)** using anti-vimentin **(a)**, anti-PDGFRα **(b)**, anti-FSP1 **(c)**, anti-cytokeratin 18 **(e** and **f)** antibodies. Secondary antibody anti-rabbit was used as control **(d)**. Scale bar, 50 μm. **B**. Immunocytochemical staining with CD68, CD163, and F4/80 antibodies. Fluorescent microscopy images indicating expressions of CD68, CD163, and F4/80 in macrophage/monocyte RAW264.7 cells **(a, b**, and **c)**. In CAFs and NHDFs, barely detectable expression is seen for CD68 **(d, g)**; meanwhile no expression can be detected for CD163 **(e, h)** and F4/80 **(f, i)**.

We extracted fibroblasts from human invasive mammary ductal carcinomas (n = 9) obtained from mastectomies. The tumor masses were dissociated, and various cell types were separated to obtain populations of carcinoma-associated fibroblasts (CAFs). We then verified the purity of the fibroblasts populations by immunostaining. These fibroblast populations strongly expressed fibroblastic markers such as vimentin (Figure 1A, a), PDGFRα (Figure 1A, b), and fibroblast surface protein-1 (FSP-1) (Figure 1A, c), whereas these cells were negative for cytokeratin (Figure 1A, e). Fibroblasts can be misidentified as macrophages because both cell types share antigens that are associated with antibodies targeting the monocyte/macrophage lineage. To determine whether macrophages cells do not contaminate the isolated cells prepared from breast cancer tissues, we used immunofluorescence to investigate the expression of various macrophage surface markers including F4/80, CD68 and CD163 [30]. Co-expression of CD68 and CD163, is a marker for the M2 anti-inflammatory macrophage phenotype [30]. As illustrated in Figure 1B, immunofluorescence revealed a barely detectable immunostaining of CD68 in CAFs (Figure 1B, d) and NHDFs (Figure 1B, g) meanwhile no expression can be detected for CD163 and F4/80 markers in CAFs (Figures 1B, e and f) and in NHDFs (Figures 1B, h and i), ruling out that the cells prepared from breast cancer tissue are not macrophages. The RAW264.7 cells, a partially differentiated macrophage-like monocytic cell line [31], was used as positive control, which expresses strongly CD68 (Figure 1B, a) and F4/80 (Figure 1B, c) markers with a moderate expression of CD163 marker (Figure 1B, b). In agreement with the present data, previous studies reported that fibroblasts isolated from normal skin, normal breast, and breast tumor tissue clearly expressed CD68 protein at levels comparable to macrophages [32, 33].

We also found that no more than 0.1% of the cells in each fibroblast population were positive for CD31, CD45, CD11b and CD268 (data not shown). Taken together, these observations indicate that these fibroblast populations were prepared with minimal contamination by epithelial, endothelial, or hematopoietic cells, such as leukocytes and erythrocytes.

### Characterization of CAFs as activated fibroblasts (myofibroblasts)

**Figure 2:**
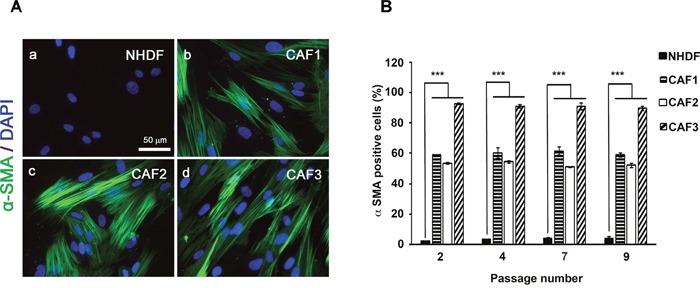
CAFs exhibit characteristic of “Myofibroblasts” **A**. NHDFs **(a)** and CAF1, 2 and 3 cells **(b, c** and **d)** were cultured in DMEM/F12 with 10% FBS and immunostained with anti-α-SMA antibody. Scale bar, 50 μm. **B**. α-SMA-positive cell counts as a fraction of total cell numbers (> 100 counted cells) were evaluated in ten independent fields from four different wells of each fibroblast type under a fluorescence microscope (*P* < 0.05).

Expression of α-smooth muscle actin (α-SMA) is a defining characteristic of myofibroblasts [34]. An increased proportion of α-SMA-positive myofibroblasts was seen in three isolated CAF populations when compared to normal human dermal fibroblasts (NHDFs) (Figures 2A, 2B). The increased α-SMA expression was largely maintained in the initially characterized CAF cells for up to nine population doublings *in vitro* (Figure 2B), indicating that isolated CAFs contain a high proportion of myofibroblasts. These results confirmed that the CAFs possessed the properties of myofibroblasts and maintained these traits without the continued presence of carcinoma cells.

### Expression of AM, CLR, RAMP2, and RAMP3 in CAFs

**Figure 3:**
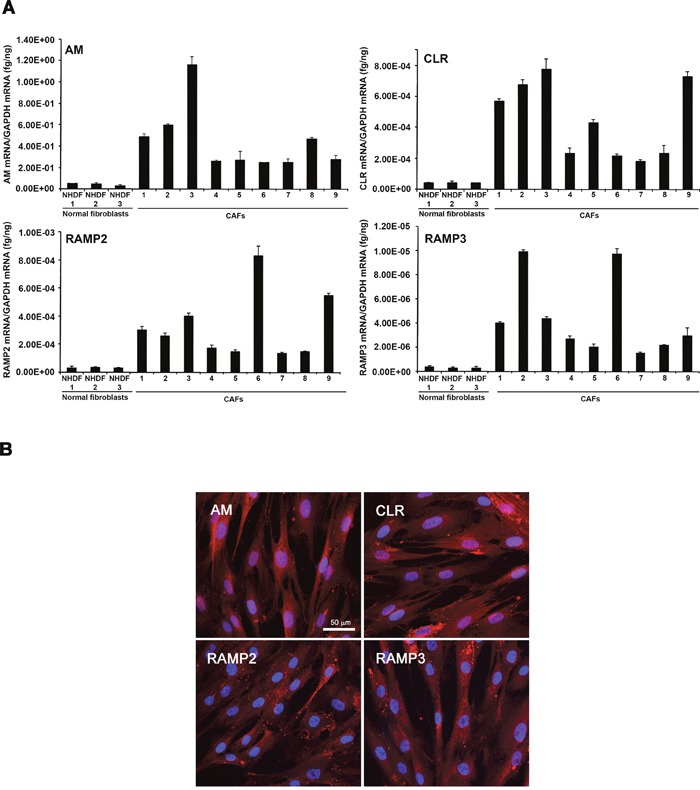
Expression of AM and its receptors in CAFs and NHDFs **A**. expression of AM, CLR, RAMP2, and RAMP3 mRNAs in NHDFs and CAFs. Total RNA (1 μg, DNA-free) prepared from NHDFs (n = 3) and CAFs (n = 9) was transcribed into cDNA and subjected to real-time quantitative reverse transcriptase-polymerase chain reaction for the estimation of the relative ratios of AM, CLR, RAMP2, and RAMP3 mRNAs to glyceraldehyde-3-phosphate dehydrogenase (GAPDH) mRNA. Each *bar* depicts the mean ± standard error of the mean of the two independent experiments from two independent preparations of total RNA from NHDFs and CAFs. **B**. immunofluorecsence for AM, CLR, RAMP2, and RAMP3 in CAFs where strong cytoplasmic staining is observed.

CAFs promote tumor formation in human breast cancers [11]. CAFs are a source of growth factors, like hepatocyte growth factor, EGF, TGF-β, and chemokines, such as CCL5 and CCL12, which are known to exert protumorigenic and prometastatic actions [35, 36]. We hypothesized that AM is one of the CAF-derived factors that might be involved in the CAFs-induced tumor formation and angiogenesis. We therefore quantified expression levels of AM mRNA in the various stromal fibroblast populations. Total RNA from NHDFs (n = 3) and CAFs (n = 9) was prepared to assess the steady-state levels of AM, CLR, RAMP2, and RAMP3 mRNA transcripts. The individual patterns of expression of AM mRNA are presented in Figure 3A. Quantification of the AM mRNA transcripts revealed 4- to 24- fold higher levels of AM mRNA in CAFs when compared to NHDFs. Among the CAFs populations, the individual pattern of expression for AM, CLR, RAMP2, and RAMP3 mRNAs was highly variable (Figure 3A). Interestingly, SDF1 mRNA demonstrated higher expression in CAFs when compared to NHDFs (Supplementary Figure 1). The increase of SDF1 expression in CAFs is in agreement with the previously reported study [11]. A clear variability of expression of AM and SDF1 mRNAs can be observed in individual CAF (Supplementary Figure 1). Omission of reverse transcriptase eliminated all signals, thus suggesting that our results were not attributable to contaminating genomic DNA.

The presence and cellular localization of AM, CLR, RAMP2, and RAMP3 in CAFs was analyzed using immunofluorescence. Representative images are shown in Figure 3B; in the images CAFs have been immunostained for AM, CLR, RAMP2 and RAMP3. Positive staining was completely abolished by pre-absorption of the antibody with 50μM synthetic peptide (not shown).

### Expression of immunoreactive AM by CAFs and NHDFs

In addition, we performed an ELISA assay on the cell extracts and medium conditioned by each CAF population as well as NHDFs. This assay indicated elevated levels of immunoreactive AM (ir-AM) in the medium conditioned by CAF1 (175 ± 10 pg/ml/24h/10^5^ cells), CAF2 (129 ± 8 pg/ml/24h/10^5^ cells), and CAF3 (190 ± 13 pg/ml/24h/10^5^ cells) cells when compared to the levels produced by the NHDFs (32 ± 5 pg/ml/24h/10^5^ cells). To determine the intracellular ir-AM levels in CAFs and NHDFs, peptide levels were measured in cell lysates. Intracellular ir-AM accumulated was in CAF1 (152 ± 8 pg/mg protein), CAF2 (137 ± 10 pg/mg protein), CAF3 (349 ± 15 pg/mg protein), and NHDFs (47 ± 5 pg/mg protein) and shows a clear increase of ir-AM content in CAFs when compared to NHDFs. These data demonstrate that ir-AM is synthesized and actively secreted by CAFs suggesting that it may function as a chemokine and/or growth factor to participate in cross talk between components of tumor microenvironment *in vivo* and/or chemoattractant of AMR^+^ circulating cells.

### AM mediate the phosphorylation of MAPK and AM blockade inhibits CAFs proliferation

**Figure 4:**
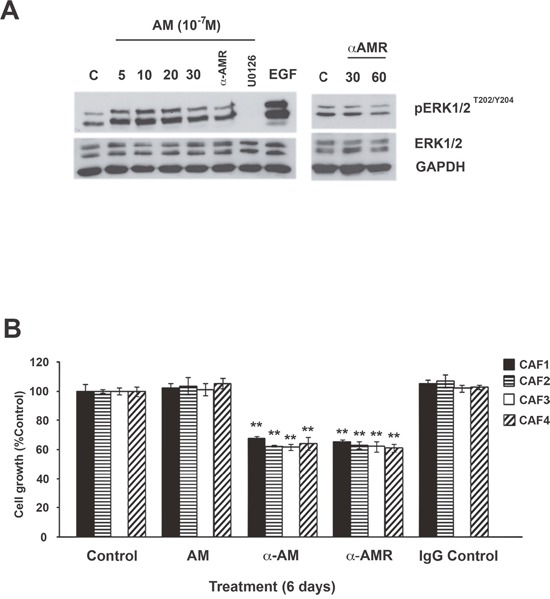
Effect of AM and AM signaling blockade on growth of CAFs *in vitro* **A**. intracellular-signaling pathway induced by AM in CAFs. CAFs were treated with AM (10^−7^ M) for the indicated amounts of time in minutes and then immunoblotted for pERK1/2 and ERK1/2. MEK inhibitor (U0126) inhibited AM induced phosphorylation of ERK (10 μM, 30 min). EGF was used as a positive control to stimulate the phosphorylation of ERK1/2. AM-induced phosphorylation of ERK1/2 is inhibited upon pre-incubation of CAFs with αAMRs for 30 min. β-GAPDH was used as a loading control. **B**. AM system blockade inhibits CAFs growth *in vitro*. Cells were seeded at a density of 2 × 10^3^ cells per well in 24 multiwell plates in the presence of medium containing 2% FBS. Cells were treated for 6 days with AM (10^−7^ M), αAM (70 μg/ml), αAMRs (70 μg/ml), or control IgG (70 μg/ml). For each treatment, six wells were prepared for MTT assay. Each *bar* represents the mean ± standard error of the mean of three independent experiments. Significant differences between the growth of cells treated with αAM, αAMRs, and that of untreated controls were determined by a one-way analysis of variance test (**p < 0.01; ***p < 0.001).

ERK and serine/threonine protein kinase (Akt) regulate cell proliferation, and both of these signaling pathways function downstream of the AM/cAMP pathway [23, 37]. Therefore, we examined the kinetics through which AM enhanced MAPK signaling. Treatment of CAFs with AM led to prolonged phosphorylation of ERK1/2 that was initially observed after 5 min of treatment and continued to increase through 20 min of treatment (Figure 4A). This increase in signaling correlated with sustained phosphorylation of ERK1/2 MAPK in CAFs (Figure 4A). Inhibition of MEK, an immediate upstream activator of ERK1/2, with U0126 (10 μM for 30 min) prevented AM-mediated activation of ERK1/2 (Figure 4A). Pre-incubation of CAFs with αAMRs prevented the stimulatory effect of AM on pERK1/2 and also decreased strongly the pERK1/2 levels observed in control cells suggesting that the endogenous AM secreted by CAFs might be involved by autocrine/paracrine loop to activate the MAPK pathway (Figure 4A). These data suggest that AM is involved in the activation of the MAPK pathway through AMR.

The activation of the MAPK pathway by AM in CAFs suggests that AM may be involved in CAFs growth through an autocrine/paracrine loop. All the CAFs showed no increase in proliferation in the presence of the maximum AM concentrations (10^−7^ M) when compared with untreated cells after 6 days treatment (Figure 4B). Consistent with an autocrine function for AM in these cells, αAM- or αAMRs-added to CAFs culture medium significantly reduced cell proliferation by as much as 40% (*p* < 0.01) when compared with cells treated with a nonspecific isotype control antibody (Figure 4B). Taken together, these observations indicate that AM is involved in the CAFs growth through AMR.

### AM secreted by CAFs contributes to the angiogenesis into *in vivo* Matrigel plug bioassays

**Figure 5:**
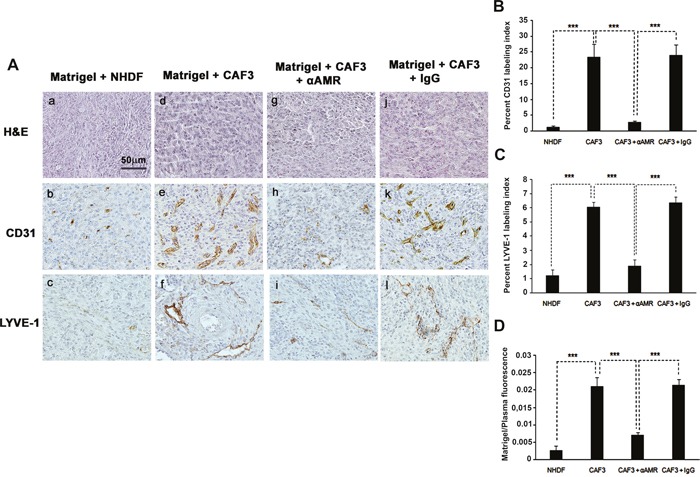
αAMR inhibit the angiogenesis and lymphangiogenesis induced-CAFs in an *in vivo* Matrigel plug bioassay **A**. C57BL/6 mice were injected s.c. at the abdominal midline with 0.4 ml of growth factor-depleted Matrigel admixed to NHDFs (1.5 × 10^6^ cells) **(a, b**, and **c)** or to CAFs (1.5 × 10^6^ cells) **(d, e**, and **f)**. αAMRs **(g, h**, and **i)** or control IgG **(j, k**, and **l)** was administered i.p. to C57BL/6 mice with Matrigel admixed to CAFs every three days, starting 24h after Matrigel injection, for 15 days. Matrigel plugs were isolated and fixed with formalin, embedded, and sectioned for immunohistochemical. Microphotographs of histochemical-stained Matrigel sections for H&E are shown **(a, d**, and **g)**. Staining of blood vessels with anti-CD31 antibody **(b, e**, and **h)** and lymphatic vessels with anti-LYVE-1 antibody **(c, f**, and **i)** of the Matrigel plugs admixed with NHDFs or CAFs is shown. Panels are representative of multiple fields from five or six plugs per group. Scale bar, 50 μm. **B & C**. quantitative assessment of the density of cells that stained positive for CD31 (B), or LYVE-1 (C) was conducted for the entire surface of the corresponding slides using CALOPIX Software. MBF_Image J 1.43U software was used for the analysis. The values shown represent the means ± standard error of the mean (***p < 0.001). **D**. after 15 days of treatment of three independent groups, mice were injected i.v. with FITC-dextran (150, 000); Matrigel plugs were removed, and the volume of new blood vessels was assessed by measurement of intravascular FITC-dextran content (normalized to FITC-dextran in the circulating plasma). Values are averages ± SE of six animals (***p < 0. 001).

We hypothesized that AM as CAFs-derived factor might be involved in the angiogenic activities of growing tumors. To this end, we used the *in vivo* Matrigel plug bioassays to assess angiogenesis in response to AM released by CAF3 in a non-inflammatory setting. Mice were injected subcutaneously (s.c.) anterior to the abdominal rectus sheath with Matrigel alone or with Matrigel admixed to CAF3, or admixed to NHDFs. *In vivo* Matrigel plug bioassays for angiogenesis revealed that plugs injected with CAF3 (Figure 5A, e, f) were significantly more vascularized than did plugs injected with NHDFs (Figure 5A, b, c) or without fibroblasts (not shown). Similar results were obtained with CAF1, CAF2, CAF6 and CAF8 (not shown). Importantly, these results demonstrate that CAFs are not only critical for the recruitment of vascular cells such as vascular and lymphatic endothelial cells and pericytes to enhance angiogenesis and lymphangiogenesis, but also that they can directly mediate these effects in the absence of tumor cells. Therefore, we hypothesized that AM secreted by CAF3 might be involved in the vascularization that occured in the *in vivo* Matrigel plug bioassays injected with CAF3.

To demonstrate that AM secreted by CAF3 is involved in the promotion of the vascular and lymphatic channels, we used treatment with αAMRs to inhibit recruitment of circulating AMR-positive cells as reported previously [25]. Matrigel plugs supplemented with CAF3 were injected s.c. into C57BL/6 mice forming semisolid plugs. Twenty-four hours later, mice were treated by intra-peritoneal (i.p.) injection with αAMRs or control IgG at 350 μg every three days for a total of 15 days. Treatment of the animals with αAMRs induced a clear decrease of the angiogenesis and lymphangiogenesis in the plugs injected with CAF3 (Figure 5A, h, i). No effect on the vascular and lymphatic channels can be observed in the plugs of group animals treated with control IgG (Figure 5A, k, l). Quantification of CD31-positive endothelial cells (Figure 5B) and LYVE-1 positive lymphatic endothelial cells (Figure 5C) demonstrated a marked decrease in the number of both cell types in plugs admixed with CAF3 from animals treated with αAMR compared to animals that received control IgG (*p* < 0.001; Figures 5A, 5B, and 5C). In second series of experiments, *in vivo* Matrigel plug bioassays for angiogenesis was quantitated by measuring the uptake of FITC-dextran (~150,000) into plugs before their removal from mice. The data demonstrate that the vascularization induced by the CAF3 injected into the plugs was stable and functional (Figure 5D). αAMRs treatment significantly inhibited by 60% to 75% the uptake of FITC-dextran in plugs compared to the plugs from animal treated with rabbit control IgG (Figure 5D). These data suggest that a part of angiogenesis and lymphangiogenesis revealed in plugs is due to AM secreted by CAF3.

**Figure 6:**
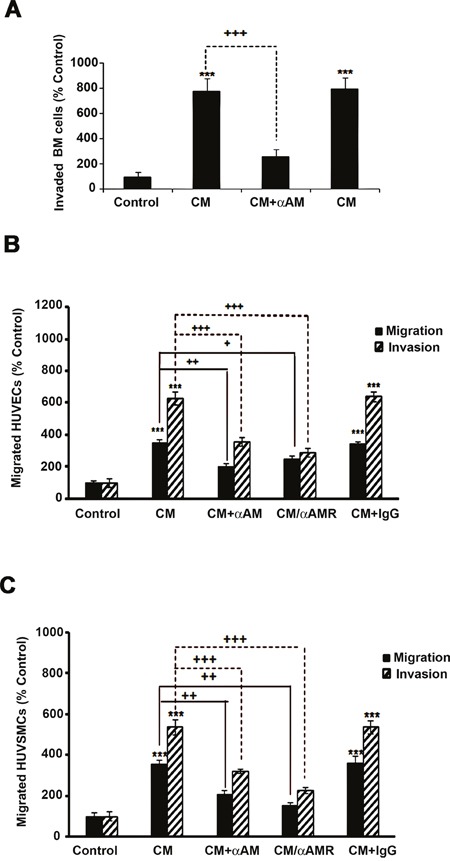
Effect of AM signaling blockade on CAF3-CM induced migration and invasion of cells *in vitro* **A, B & C**. CAF3-CM regulates migration and invasion of HUVECs and HUVSMCs and invasion of BMDCs *in vitro*. The bottom wells of all chambers were filled with CAF3-CM and the control well was filled with DMEM containing 2% FBS (control). To neutralize the ir-AM secreted in the CAF3-CM, it was pretreated for 30 minutes with αAM (70 μg/ml). Bone Marrow cells (A, 5 × 10^5^ cells), HUVECs (B, 3 × 10^4^ cells), or HUVSMCs (C, 3 × 10^4^ cells) pretreated for 30 min with 23 μg/ml each of αCLR, αRAMP2 and αRAMP3 (αAMRs), or control IgG (70 μg/ml) were placed in the upper chamber and incubated as described in the Materials and Methods. The cells that migrated were stained with DAPI and counted at 50x magnification using a microscope. Data are expressed as the number of migrated cells in 10 high-power fields, and the values represent the mean ± SEM of three independent experiments, each performed in triplicate. The asterisk (*) is used for comparison to control cells (**p < 0. 01; ***p < 0. 001) and the plus symbol (+) is used in comparison to CM-treated cells (++*p* < 0. 01; +++*p* < 0. 001).

**Figure 7:**
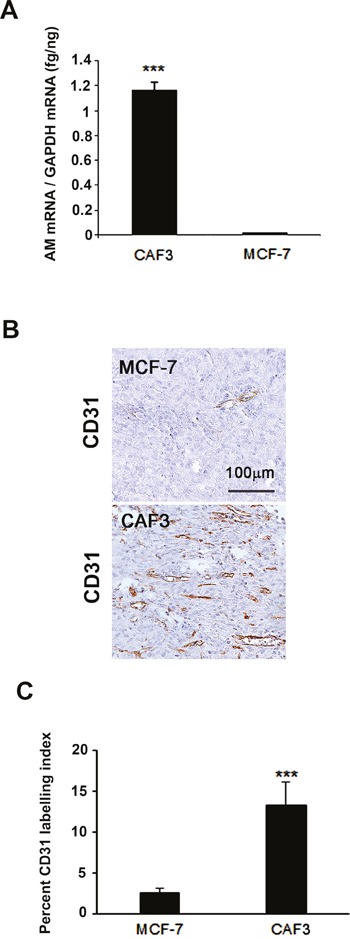
MCF-7 cells showed barely detectable angiogenesis compared to CAFs in an *in vivo* Matrigel plug bioassay **A**. Expression of AM mRNA in MCF-7 cells and CAF3. Total RNA (1 μg, DNA-free) prepared from MCF-7 cells and CAF3 was transcribed into cDNA and subjected to RT-qPCR as described in the Figure 3A (***p < 0.001). **B**. C57BL/6 mice were injected s.c. at the abdominal midline with 0.4 ml of growth factor-depleted Matrigel admixed to MCF-7 (1.5 × 10^6^ cells) or to CAFs (1.5 × 10^6^ cells) for 15 days. Matrigel plugs were processed as described in Figure 5. Staining of blood vessels with anti-CD31 antibody of the Matrigel plugs admixed with MCF-7 cells or CAFs is shown. Panels are representative of multiple fields from five or six plugs per group. Scale bar, 50 μm. **C**. quantitative assessment of the density of cells that stained positive for CD31 was conducted for the entire surface of the corresponding slides using CALOPIX Software. MBF_Image J 1.43U software was used for the analysis. The values shown represent the means ± standard error of the mean (***p < 0.001).

To strengthen our findings, we therefore tested the possibility that AM secreted by CAFs might be involved into the recruitment of endothelial cells and pericytes to foster a functional and stabilized angiogenesis. We had shown that AM induces recruitment of different cell types such as endothelial-like cells, pericytes, and macrophages/monocytes in an *in vivo* Matrigel plug bioassays [25]. AM receptors are expressed in cultured primary HUVECs [38] and HUVSMCs [23], suggesting that these cells could be recruited by AM secreted by CAFs to assist neo-vessels formation during tumor growth. The migration and invasion assays demonstrate that CAF3-conditioned medium (CAF3-CM) promoted invasion of bone marrow-derived cells (BMDCs), migration and invasion of HUVECs and HUVSMCs in Transwell assay (Figures 6A, 6B, and 6C). Neutralization of CAF3-CM with a function-blocking antibody to AM (αAM) or pre-incubation of cells with αAMRs significantly inhibited the stimulating effects of CAF3-CM on migration and invasion (Figures 6A, 6B, and 6C). These data strongly suggest that AM must be one of the CAF-derived factors responsible for endothelial cells, pericytes/smooth-muscle cells and BMDCs recruitment to promote a stabilized and functional angiogenesis. Importantly, *in vivo* Matrigel plug bioassays for angiogenesis revealed that plugs injected with CAF3 were significantly vascularized than did plugs injected with a human breast cancer cell line MCF-7 which express low basal levels of AM mRNA (Figures 7A, 7B, and 7C).

### AM blockade inhibits the growth of MCF-7 cells/CAF3- tumor xenografts *in vivo*

**Figure 8:**
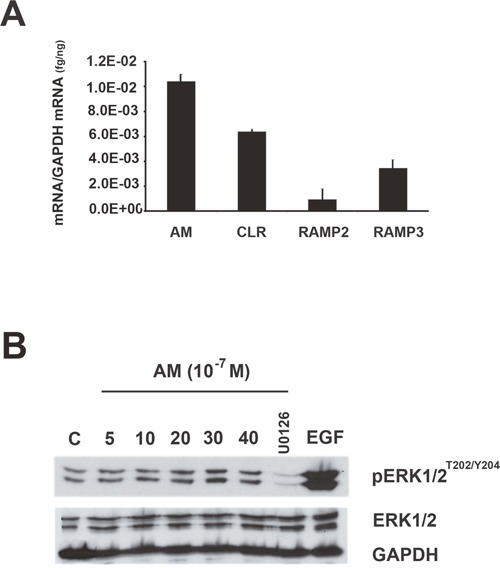
MAPK pathway is activated by AM in MCF7 cells **A**. expression of AM, CLR, RAMP2, and RAMP3 mRNAs in MCF7 cells. Total RNA (1 μg, DNA-free) prepared from MCF7 cells was transcribed into cDNA and subjected to real-time quantitative reverse transcriptase-polymerase chain reaction for the estimation of the relative ratios of AM, CLR, RAMP2, and RAMP3 mRNAs to glyceraldehyde-3-phosphate dehydrogenase (GAPDH) mRNA. Each *bar* depicts the mean ± standard error of the mean of the three independent experiments from three independent preparations of total RNA from MCF7 cells. **B**. intracellular-signaling pathway induced by AM in MCF7 cells. MCF cells were treated with AM (10^−7^ M) for the indicated amounts of time in minutes and then immunoblotted for pERK1/2 and ERK1/2. MEK inhibitor (U0126) inhibited AM induced phosphorylation of ERK (10 μM, 30 min). EGF was used as a positive control to stimulate the phosphorylation of ERK1/2. β-GAPDH was used as a loading control.

To assess the role of AM in CAFs function, we adopted a well-established bioassay, involving co-inoculation with transformed epithelial cells into a heterotopic site, where the effects of myofibroblasts on tumor growth could be assessed [6, 11]. We choose the MCF7 cells since they express barely detectable levels of AM mRNA (Figure 8A) and ir-AM (< 6 pg/mg protein) to evaluate better the role of AM expressed by the myofibroblasts in tumor growth *in vivo* when CAFs and MCF7 cells are co-injected to nude mice. Interestingly, MCF7 cells express AMR suggesting that it might be sensitive to AM (Figure 8A). In fact, treatment of MCF7 cells with AM led to phosphorylation of ERK1/2 that was observed after 20 min of treatment (Figure 8B). Inhibition of MEK with U0126 (10 μM for 30 min) prevented the stimulatory effect of AM on pERK1/2 (Figure 8B). These data suggest that AM could be one of the factors secreted by tumor microenvironment to cross talk with breast cancer cells *in vivo*.

**Figure 9:**
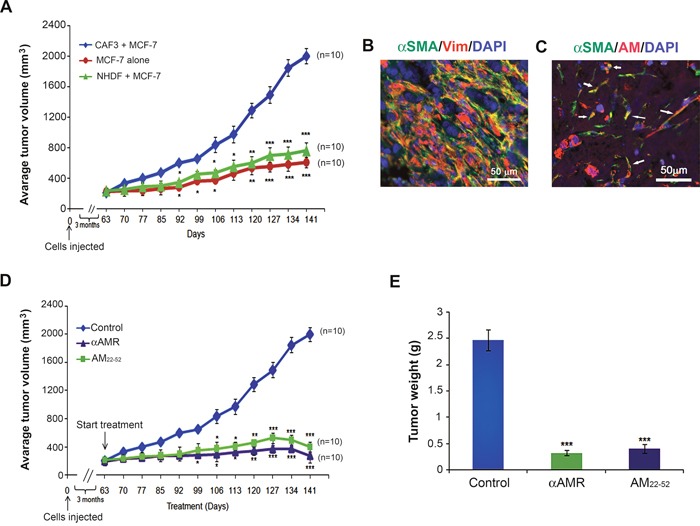
Enhanced tumor growth kinetics of MCF-7 breast cancer cells comingled with CAF3 **A**. MCF-7 cells (1 × 10^6^ cells) were injected alone or coinjected with myofibroblasts (CAF3) (3 × 10^6^ cells) subcutaneously into nude mice. Tumor volume was ploted in indicated days (**p < 0.01; ***p < 0.001). **B & C**. MCF-7 cells/CAF3 xenograft sections were immunostained with anti-α-SMA and anti-vimentin (B) with a merged view shown, or anti-AM and anti-α-SMA antibodies (C). AM^+^ α-SMA^+^ myofibroblasts are shown in a merged view (C, arrows). Scale bar, 50 μM. **D**. AM signaling blockade inhibited the growth of MCF-7 cells/CAF3 xenografts *in vivo*. MCF-7 cells (1 × 10^6^ cells) admixed to CAF3 (3 × 10^6^ cells) were injected subcutaneously into the flanks of athymic nude mice (6 weeks old) (n = 10 in each group). Mice with tumor volume averaging ~200 mm^3^ received i.p. injections of αAMRs (12 mg/kg) every 3 days or AM_22-52_ peptide (50 μg/mouse) daily. Control mice were treated with 12 mg/kg of nonspecific isotype control IgG. Tumor size was measured every 3 days, and significant differences between the animals treated with αAMRs and AM_22-52_ and those treated with control IgG were determined by a one-way analysis of variance test (**p < 0.01; ***p < 0.001). **E**. tumors were weighed immediately after excision and the average tumor weight is indicated as the mean ± SEM (n = 10).

To investigate the role of AM expressed by CAFs in tumor growth of breast cancer cells *in vivo*, MCF-7 cells were injected s.c. in immunodeficient mice either alone, or admixed with CAF3. Tumors co-injected with CAF3 grew significantly faster and were larger than tumors in mice injected only with MCF-7 cells (Figure 9A) indicating a potent increased tumor cell proliferation in the presence of CAF3. We hypothesized that breast CAFs might support tumor growth in part through the stimulation of angiogenesis, via the secretion of AM. Interestingly, the tumors in mice co-injected with NHDFs had a phenotype not different from the one obtained with the MCF-7 alone (Figure 9A).

Remarkably, the co-inoculated CAF3 survived in large numbers in tumors together with carcinoma cells for periods of up to 29 weeks after injection, as determined by immunohistochemistry using an antibody specific for human vimentin (Figure 9B) and α-SMA (Figure 9B), which MCF-7 cells fail to express. It is of interest to observe that CAF3 demonstrates expression of AM in xenografts *in vivo* (Figure 9C, arrows) which demonstrates that CAF3 maintained the expression of AM *in vitro* as well as *in vivo*.

To evaluate the functional role of AM in MCF-7/CAF3 xenografts growth, we investigated the effects of inhibition of AM signaling on tumor xenografts. To assess the potential therapeutic value of αAMRs and AM antagonist (AM_22-52_), athymic nude mice bearing established MCF-7/CAF3 xenografts (~200 mm^3^) were treated with αAMRs, AM_22-52_, or control IgG. Treatment was administered by i.p daily injection for AM_22-52_ (50 μg/mouse), and every 3 days for αAMRs (12 mg/kg) and control IgG (12 mg/kg) as reported previously [23, 24]. To monitor tumor growth, tumor volume was measured throughout the treatment period. The growth of the MCF-7/CAF3 xenografts was significantly reduced by αAMRs and AM_22-52_ treatments compared with that in the control group (Figure 9D). After 12 weeks treatment period, animals were sacrificed, and tumor size was assessed. The mean tumor weights in the animals treated with control IgG, αAMRs, and AM_22-52_ were 2.5 ± 0.4 g, 0.4 ± 0.10 g, and 0.5 ± 0.15 g, respectively (Figure 9E).

### AM blockade impairs tumor angiogenesis and induces apoptosis

**Figure 10:**
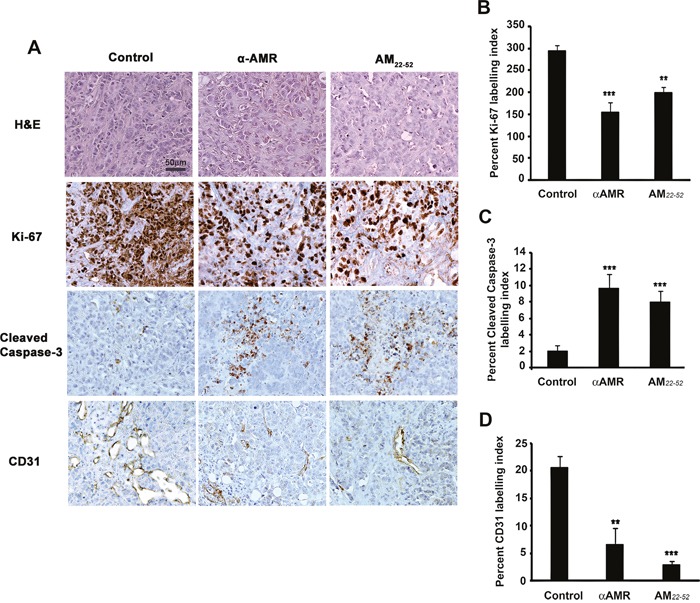
AM blockade induces apoptosis and impairs angiogenesis in MCF-7/CAFs tumor xenografts **A**. representative images of tumors from the animals treated with control IgG, αAMRs, and AM_22-52_. Tumor sections were stained with H&E, Ki-67, cleaved caspase-3, and CD31. Cleaved caspase-3- and Ki-67-positive cells are shown; they were analyzed on the basis of 10 magnification fields (400x) per section. Immunohistochemical staining of the endothelial cell surface marker CD31 was used to determine the microvessel density. Quantitative assessment of the density of cells that stained positive for Ki-67 **B**. cleaved caspase-3 **C**. or CD31 **D**. was conducted for the entire surface of the corresponding slides using CALOPIX Software. MBF_Image J 1.43U software was used for analysis. The values shown represent the mean ± SEM (** *p* < 0. 01; *** *p* < 0. 001).

Immunohistochemical staining performed on the tumor xenografts demonstrated significant differences in the Ki-67 labeling index between the animals treated with αAMRs, AM_22-52_, and control IgG (Figures 10A and 10B). Significantly higher numbers of cleaved caspase-3-positive cells were observed in tumors from the animals treated with αAMRs and AM_22-52_ (Figures 10A and 10C). MCF-7/CAF3 xenografts from IgG-control-treated animals were significantly more vascularized, as assessed by immunostaining for the endothelial cell marker CD31 (Figure 10A). Consistent with our hypothesis that AM signaling inhibition would result in a decrease in angiogenesis, immunohistochemical staining for CD31 demonstrated that tumor vascularization is deeply disrupted in tumors from animals treated with αAMRs and AM_22-52_ (Figure 10A). Quantification of CD31-positive endothelial cells demonstrated a clear decrease in tumors from animals treated with αAMRs and AM_22-52_ compared with the levels in control tumors (Figures 10A and 10D).

## DISCUSSION

CAFs, myofibroblast-rich cell populations extracted from human carcinomas maintain an ability to promote tumorigenesis. These cells, passaged for 10 passages doubling *in vitro* without ongoing interaction with carcinoma cells, retained their ability to promote tumor growth when co-injected with carcinoma cells into immunodeficient mice [39, 40]. However, the molecular mechanisms underlying their tumor-promoting ability are poorly understood. Some have reported the importance of somatic genetic alterations in forming the tumor-promoting stroma, yet their existence remains controversial [41–43].

In the present experiments, we show that (i) The CAFs exhibit increased α-SMA expression that is indicative of myofibroblasts. (ii) Immunofluorescence with macrophages markers CD68, CD163 and F4/80 revealed that CD68 was also cross-reactive but with less intensity in fibroblasts as reported previously [33]. Both antibodies against monocytes/macrophage markers, F4/80 and CD163 do not stained CAFs populations, ruling out the presence of macrophages and identified the isolated cells as cancer-associated fibroblasts (CAFs). (iii) Myofibroblasts produce increased levels of AM mRNA as well as ir-AM when compared to normal fibroblasts. (iv) αAM and αAMR could inhibit the basal levels of CAFs proliferation *in vitro*, which is consistent with the fact that AM can act in an autocrine/paracrine manner to induce CAFs proliferation and increases activation of the MAPK pathway. The presence of an autocrine loop suggests that the foci of AM-producing cells in tumor tissue could stimulate cells expressing AM receptors through autocrine/paracrine mechanisms. (v) Myofibroblasts extracted from within invasive human breast cancer masses are more competent than normal fibroblasts in enhancing a stable vascularization in *in vivo* Matrigel plug bioassay for angiogenesis. (vi) ir-AM secreted by the myofibroblasts is responsible of induction of migration and invasion of endothelial cells, pericytes, and BMDCs *in vitro* and *in vivo*, thereby boosting tumor angiogenesis. (vii) We demonstrated that some CAFs cultures expressed both AM and SDF-1 mRNAs. It will be of interest to understand whether AM and SDF-1 peptides act in concert or separately to promote tumor growth when both are relatively well expressed in CAFs.

We demonstrated that CAFs admixed with breast cancer cells, enhanced angiogenesis both in heterotopic MCF-7 tumors and in an *in vivo* Matrigel plug bioassay lacking cancer cells. The ability of CAFs to influence tumor growth was partly dependent on their ability to induce angiogenesis by CAF-derived AM and recruitment of BMDCs. On the basis of results from our study as well as those from previous report [44], we conclude that AM is an angiogenic and tumor promoting factor that is secreted by tumor cells and stromal cells such as myofibroblasts in breast cancer tumors.

The blockade of the AM receptors by systemic administration of αAMRs or AM_22-52_, inhibits angiogenesis in an *in vivo* model and the growth of admixed MCF-7 cells/CAF3 xenografts. We demonstrated that addition of CAF3 to Matrigel plug bioassay *in vivo* significantly enhanced plug neovascularization, which was effectively inhibited by systemic injection of αAMRs. This blocking effect was confirmed on tumor xenografts growth *in vivo*. In fact, the treatment of admixed MCF-7 cells/CAF3 xenograft-bearing mice with αAMRs or AM_22-52_ consistently resulted in tumor regression, suggesting that the tumor is most susceptible to αAMRs and AM_22-52_ therapies. CAF3-treated with αAM or αAMRs showed a growth inhibition *in vitro* suggesting that the growth inhibition *in vivo* might be due to the effect of αAMRs and AM_22-52_ on tumor vasculature and CAFs growth *in vivo*. The immunohistochemical analysis of admixed tumors xenografts derived from animals treated with αAMRs or AM_22-52_ showed a clear decrease in microvessel density, with a 65% to 80% reduction in endothelial cells and pericytes within the tumor, which is consistent with the role of AM in endothelial cell and pericyte survival and recruitment. Interestingly, the density of vessels with lumen decreased dramatically. The loss of microvessels within αAMRs or AM_22-52_-treated tumors suggests that AM stimulation of CLR/RAMP2/RAMP3-expressing tumor vasculature acts as a survival mechanism for proliferating tumor endothelium. In agreement with previous studies, these data demonstrate that AM secreted by CAFs could play a role to foster a stabilized and functional neovascularization in growing tumors [25, 38, 45]. Recently, we showed that the blockade of AM signaling selectively targets unstable tumor neovessels through rapid disengagement of the VE-cadherin/β-catenin complex, destabilization of the cytoskeleton organization of endothelial cells, and subsequent apoptosis-mediated cell death [46].

The data reported in the present study echo findings of others demonstrating that stromal fibroblasts isolated from human prostate carcinomas have an increased ability to foster tumor formation when compared to normal prostatic fibroblasts [6]. In 1999, the groups of Tlsty and Cunha demonstrated a striking tumor-promoting property of stromal fibroblasts extracted from human prostate carcinomas when these were compared with control normal fibroblasts isolated from the noncancerous prostate gland [6]. Such fibroblasts have been shown to regulate carcinoma cell growth, differentiation, and tumorigenesis, either in a positive or negative fashion [47–49].

CAFs exhibit increased levels of secretory molecules that include growth factors and chemokines such as VEGFA, HGF, PDGF, TNF, IL-6, IL-8 and SDF-1 [for review see ref 50]. CAFs have been shown to promote tumor growth by directly stimulating tumor cell proliferation and by enhancing angiogenesis [3, 10, 11]. Fibroblasts are also key players in wound healing [51, 52], mediating extracellular matrix remodeling and generation of contractile forces. It has also been suggested that fibroblasts mediate the transition from acute to chronic inflammation by inappropriately providing recruitment, survival, and retention signals to infiltrating leucocytes, thus inhibiting the normal resolution of inflammation [53–55]. Previous studies have reported that fibroblasts are a source of cytokines or chemokines or both in tumors [56–58]. Moreover, a study-using laser capture microdissection revealed CXCL12 and CXCL14 to be upregulated in stroma of prostate and basal cell carcinomas [59, 60]. The ability of CAFs to influence tumor growth was partly dependent on their ability to induce angiogenesis by CAF-derived SDF-1 (also known as CXCL12) and recruitment of bone marrow-derived endothelial cells [11] or by CAF-derived PDGF-C, a member of the PDGF family [12] or secreting proangiogenic factors [61].

The notion of targeting CAFs to inhibit tumor growth and progression is attractive due to the increasing identification of stimulatory factors derived from the tumor stroma. Targeting of stromal fibroblast effector functions by drugs inhibiting the action of osteopontin, CXCL12, CXCL14, FGF-2, PDGF-C, AM, and many more, singularly or in combination, holds promise of producing therapeutic efficacy. In addition, approaches to prevent the recruitment and phenotypic conversion of CAFs are highly warranted.

## MATERIALS AND METHODS

### Cell culture

The human breast cancer cell line MCF7 and the partially differentiated monocyte-macrophage cell line RAW 264.7 were obtained from the American Type Culture Collection (ATCC, Rockville, MD, USA). The cells were grown in a humidified atmosphere at 37°C and 5% CO_2_ in DMEM (Lonza BioWhitaker, France) supplemented with L-glutamine (2 mM) and 5% heat-inactivated fetal calf serum (FCS) (Lonza) and in absence of antibiotics. HUVECs and HUVSMCs (Lonza) were cultured in EGM-2 medium (Lonza) containing 2% FBS and M199 medium (Invitrogen Life Technologies Inc.) containing 20% FBS, respectively, in humidified atmosphere at 37°C with air/5% CO_2_. HUVECs and HUVSMCs monolayers from passages 2–4 were used in these studies.

### Isolation and culture of cancer–associated fibroblasts

Cancer–associated fibroblasts (CAFs) were isolated from human breast tumor biopsies as previously described [62]. Tissues were digested with F15 medium with 0.037% hyaluronidase (sigma), 1 mg/ml collagenase/dispase (Roche), 1% FBS, and antibiotics for 1-2 hr at 37°C. Dissociated cells were filtered through both 100 μm and 40 μm filters. Washed filtrates were plated onto 1% gelatin-coated plates and cultured in 10% FBS-containing DMEM/F12 medium maintained at 37°C in a humid atmosphere of 95% air/ 5% CO_2_. After 48 hours incubation, suspension tumor cells were removed after vigorous washing. After 2-3 passages, an apparently pure fibroblast population was obtained. All CAFs used in this study had undergone fewer than ten population doublings in culture. Normal human dermal fibroblasts (NHDFs) (Promocell) were cultured in promocell growth medium (Promocell).

### Immunostaining of the human fibroblasts

Primary cultured fibroblasts were examined by immunofluorescence using anti-cytokeratin-18 (Sigma), human specific anti-vimentin (V9; Novocastra laboratories, Ltd., UK), anti-fibroblast surface protein (1B10; Sigma), anti-human CD31 (Santa Cruz, California), anti-α–SMA (1A4; Dako, Denmark), anti-PDGFRα (Dako), anti-human CD68 (1/100, BD Pharmingen™), anti-human CD163 (1/100, Bio-Rad), and anti-human F4/80 (1/50, Invitrogen) antibodies. The antibodies anti-AM, anti-CLR, anti-RAMP2, and anti-RAMP3 were developed and characterized in the laboratory.

### Immunoassay for human AM

CAFs (1.5 × 10^6^) and NHDFs (1.5 × 10^6^) were cultured in DMEM/F12 medium for 48 hours. The immunoreactive AM in cell extracts as well as in medium was measured using a commercially available AM ELISA kit (Euromedex, Strasbourg, France).

### RNA preparation and real-time quantitative RT-PCR

Total RNA was prepared from CAFs, NHDFs, and MCF-7 cells and reverse transcribed to cDNA as described [63]. Human AM, CLR, RAMP2, RAMP3 and GAPDH mRNAs [58] and human stromal-derived factor (SDF1) mRNA (forward primer 5’-CGATTCTTCGAAAGCCATGT-3’ and reverse primer 5’-CTTGCTTGTTGTTGTTCTTCAGC-3’) were amplified, detected and quantitated in real-time using LC480 PCR system (Roche Diagnostics, Meylan, France) as described [63, 64].

### Western blot analysis

Cell extracts were prepared and immunoblotted for phospho-ERK1/2, and ERK1/2 using the MAPK-phospho-ERK1/2 pathway sampler kit (Cell signaling Technology, Inc.) as previously described [23]. Antibody signals were revealed using an enhanced chemiluminescence kit (ECL kit, Invitrogen Life Technologies Inc.).

### Cell proliferation assay

AM (10^−7^ M), rabbit anti-human AM (αAM; 70 μg/ml), and anti-human AM receptor (αCLR, αRAMP2 and αRAMP3) (70 μg/ml) neutralizing antibodies (purified IgG) previously developed in house [21, 38] were added daily to the culture to evaluate their effects on cell proliferation. After six days treatment, the effects were examined by 3-(4, 5-dimethylthiazol-2, 5-diphenyltetrazolium bromide) (MTT) assay (Promega, Lyon, France).

### Transwell migration and invasion assays

A modified Boyden chamber assay was used to analyze migration and chemoinvasion of murine bone marrow derived cells (BMDCs), HUVECs, or HUVSMCs as described previously [38, 64].

### *In vivo* Matrigel plugs studies and analysis

Female C57BL/6 mice were injected s.c. above the rectus abdominus with 600 μl of Matrigel (Becton-Dickinson, Le Pont de Claix, France), admixed to NHDFs (1.5 × 10^6^) or CAFs (1.5 × 10^6^) in 50 μl of PBS or alone as a negative control. Twenty-four hours later, mice injected with Matrigel combined with CAFs were randomized into two groups and treated i.p. with αAMRs (330 μg) or preimmune serum (purified IgG, 330 μg) every three days. Two weeks later, animals were sacrificed, and the Matrigel plugs were dissected and fixed in 4% paraformaldehyde (PFA) for histological analysis. Immunohistochemical analysis was performed on paraffin-embedded sections using the Vectastain Elite ABC Universal kit (Vector Laboratories, Burlingame, CA) with antibodies from Dako Inc. (Glostrup, Denmark) for CD31 (1:20) and LYVE-1 (1:100).

The second group of animals was injected systemically into the lateral tail vein with 100 mg/kg FITC-dextran solution (molecular weight ~ 150,000; Sigma Chemical Co., Lyon, France) and allowed to circulate for 25-30 min. Before mice sacrifice, blood samples were collected by cardiac puncture and plasma was separated. The Matrigel plugs were resected, placed into tubes containing Dispase reagent (Thermo-Fisher, Cergy-Pontoise, France), and homogenized. After centrifugation, the supernatant was saved for analysis of fluorescence. Fluorescence readings were obtained on an FL600 fluorescence plate reader (BioTek). Angiogenic response was expressed as a ratio of Matrigel plug fluorescence/plasma fluorescence.

### *In vivo* tumor growth assessment

MCF-7 cells (1 × 10^6^) alone (n = 10) or admixed with CAFs (3 × 10^6^) (n = 30) were injected subcutaneously into the right flank of female nude mice. Tumors sizes were determined with a dial-caliper measurements, and the tumor volume was calculated as width × length × height × 0.5236 (for ellipsoid form). Mice with tumor volume greater than 2,000 mm^3^ were sacrificed in accordance with Aix-Marseille University Animal Rights Committee guidelines. When the tumors were ~200 mm^3^ in size, animals were randomly divided into three groups. One group (*n* = 10) received intraperitoneal (i.p.) injection of αAMRs (12 mg/kg purified IgG in 200 μl PBS) every 3 days; the second group (n = 10) received 50 μg of AM antagonist AM_22-52_ daily as described previously [24]; and the third group (n = 10) received an irrelevant antibody (IgG of the same isotype). The αAMRs were characterized as described [25] and all IgG preparations were tested for endotoxin using the Pyrogent plus Limilus ameboycote lysate kit (Lonza). All antibody preparations used in animal studies contained < 1.25 U/ml endotoxin. Tumors sizes were measured every 3 days, and mice were sacrificed at 29 weeks after injection. Tumors were embedded in Paraffin for pathologic studies and immunohistochemistry.

### Immunohistochemical staining

Sections (6 μm) were cut from formalin-fixed paraffin-embedded xenografts. Immunohistochemistry was performed using the Vectastain Elite ABC Universal kit (Vector Laboratories, Burlingame, CA) as described previously [23, 64]. Antibodies against CD31 (1:20, Dianova), Ki-67 nuclear antigen (1:100; Dako), and cleaved caspase-3 (1:100; BD Pharmingen™) were used for the analysis. For each marker, whole-surface staining was quantified using Image J Software (NIH, Bethesda, USA).

### Tumor vascular density

Quantitation of vessel count was performed as previously described [65]. The blood vessels were counted randomly from non-necrotic areas in each tumor section in an x200 microscope field, on CD31-stained tissue sections.

### Statistical analysis

Data are expressed as means ±SEM from at least three independent experiments. One-way analysisof variance (ANOVA) or Fisher's PLSD test (Statview 512; Brain Power Inc., Calabasas, CA, USA) was used for statistical analysis. Differences were considered significant at values of *p* < 0.05.

## SUPPLEMENTARY MATERIALS FIGURES AND TABLES


