# Supplementary material for: Stromal fibroblasts present in breast carcinomas promote tumor growth and angiogenesis through adrenomedullin secretion
Source: Oncotarget. 2017 Feb 2;8(9):15744–62. doi: 10.18632/oncotarget.14999 (PMC5362520; doi:10.18632/oncotarget.14999)
Supplement: Supplementary file 1 [file oncotarget-08-15744-s001.pdf]

Stromal fibroblasts present in breast carcinomas promote tumor growth and angiogenesis through adrenomedullin secretion

SUPPLEMENTARY FIGURE

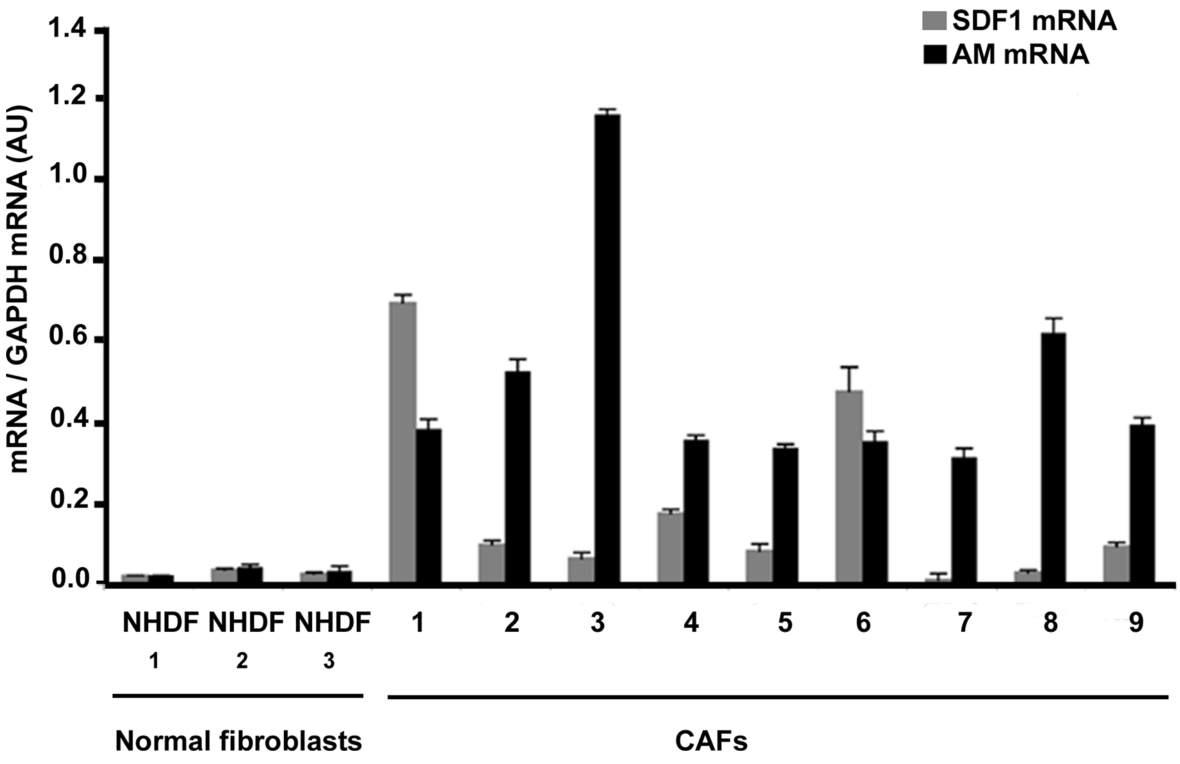

Supplementary Figure 1: Expression of AM and SDF1 mRNAs in CAFs and NHDFs. Expression of AM and SDF1 mRNAs in NHDFs and CAFs. Total RNA (1 µg, DNA-free) prepared from NHDFs (n = 3) and CAFs (n = 9) was transcribed into cDNA and subjected to real-time quantitative reverse transcriptase-polymerase chain reaction for the estimation of the relative ratios of AM and SDF1 mRNAs to glyceraldehyde-3-phosphate dehydrogenase (GAPDH) mRNA. Each *bar* depicts the mean ± standard error of the mean of the two independent experiments from two independent preparations of total RNA from NHDFs and CAFs.
